# Editorial: The glutamate hypothesis of mood disorders: Neuroplasticity processes, clinical features, treatment perspectives

**DOI:** 10.3389/fpsyt.2022.1054887

**Published:** 2022-11-29

**Authors:** Riccardo Guglielmo, Rocco de Filippis, Sami Ouanes, Gregor Hasler

**Affiliations:** ^1^Department of Neuroscience, Institute of Psychiatry, Catholic University Medical School of Rome, Rome, Italy; ^2^Psychiatry Research Unit, Fribourg Network for Mental Health (RFSM), University of Fribourg, Fribourg, Switzerland; ^3^Institute of Psychopathology, Rome, Italy; ^4^Department of Psychiatry, Hamad Medical Corporation, Doha, Qatar

**Keywords:** mood disorders, glutamate hypothesis, serotonin hypothesis, ketamine, neuroplasticity

The monoamine hypothesis has dominated the research on the pathophysiology of mood disorders as well as the development of therapeutic drugs by over half a century. The monoamine theory proposes that serotonin, norepinephrine, and dopamine deficiencies are responsible for the occurrence of depressive symptoms. Early on, monoamine depletion studies demonstrated that this simple theory is incorrect since serotonin and catecholamine depletion did not consistently include depressive symptoms (1). Only in subjects with remitted mood disorders, some studies showed a relationship between monoamine levels and depressive psychopathology (2). In line, further studies into monoaminergic dysfunction in mood disorders have also yielded inconsistent results, including monoamine receptor theories of depression (3). As a consequence, we have to assume that conventional antidepressants that increase monoamine levels in the synaptic cleft do not target a primary dysfunction in mood disorders but rather exert a non-specific effect that mitigates stress and anxiety symptoms without having profound effects on core aspect of depression such as anhedonia, stress-induced reductions in neuroplasticity and neuronal dystrophy. Although this non-specific monoaminergic effect has exponentially improved the treatment of mood disorders, only 30% of patients with major depressive disorder (MDD) who receive an adequate treatment experience full remission (4) and up to 46% of patients suffering from depression do not respond to first-line treatment and develop a treatment resistant depression (TRD) (5). TRD was defined as an inadequate response to two or more antidepressants after adequate dosing and duration (5).

Nowadays a change of perspective is taking place (Figure 1). The glutamate system is increasingly implicated in the pathophysiology of mood disorders. The evidence spans from animal, post-mortem, imaging, pharmacological and genome-wide association studies in MDD and bipolar disorder (BD). These disorders have been recently re-conceptualized as a synaptic plasticity-related disorders rather than simply as deficits or excesses in individual neurotransmitters. A paradigm shift from a monoamine hypothesis to a neuroplasticity hypothesis focused on glutamate may represent a substantial advancement in the research for new drugs and therapies. In this context, research into the neuroprotective effects of mood stabilizers and the neuroplasticity effects of glutamatergic psychedelics is becoming increasingly important. The neuroplasticity hypothesis of mood disorders stems from the evidence that acute and chronic stress and depression itself lead to glutamate release dysregulation, impaired glutamate cycling and metabolism, and glutamatergic excitotoxity. This complex dysregulation induces neuronal dystrophy and overall synaptic depression in the prefrontal cortex (PFC) and the hippocampus while other cerebral regions, like the amygdala, show changes consistent with neuronal hypertrophy and synaptic potentiation (6). The subsequent fronto-limbic synaptic disconnection is critical to the progress and treatment of mood disorders. The interplay of glial and glutamatergic system along with neuroinflammation and stress-induced changes involving these systems among others may be deeply involved in the pathogenesis of mood disorders. In this context, the kynurenine (KYN) pathway is becoming increasingly important (7, 8). Some of these questions are investigated by studies of this Research Topic.

**Figure 1:**
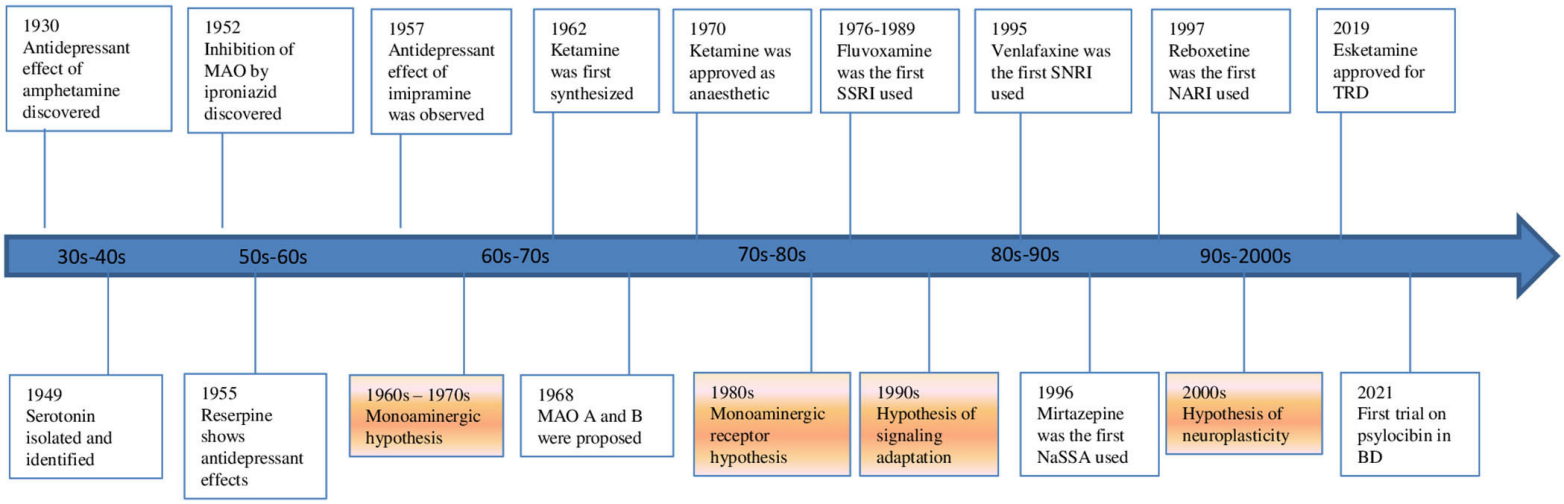
Evolution of hypothesis and antidepressants in mood disorders.

Kang et al. reviewed the neuroplasticity mechanisms behind the rapid antidepressant effects of ketamine in MDD and BD, including TRD. They focused particularly on the immediate molecular cascades that may mediate ketamine's rapid antidepressant effects both *in vivo* and *in vitro*, in animals as well as in humans. The results of their systematic review confirmed the glutamatergic activity of ketamine as the main mechanism of action behind its rapid antidepressant effect. They found that molecular neuroplastic changes that occur rapidly after exposure to ketamine encompass an increase in molecules involved in modulating neuroplasticity, particularly glutamate, AMPA receptors (AMPARs), the mammalian target of rapamycin (mTOR), brain-derived neurotrophic factor (BDNF), tropomyosin receptor kinase (TrkB), nerve growth factor inducible (VGF), eukaryotic elongation factor-2 kinase (eEF2K), P70 ribosomal S6 kinase (p70S6K), glycogen synthase kinase 3 (GSK-3), insulin-like growth factor 2 (IGF2), extracellular signal-regulated kinase (Erk), and microRNAs.

In their neuroimaging review, Demchenko et al. discussed brain imaging studies that examine brain connectivity features associated with rapid antidepressant response in MDD patients treated with glutamatergic pharmacotherapies, with a focus on the intrinsic connectivity networks (ICNs). They suggest a neuroanatomical framework for studying glutamatergic ICNs in depression as this can improve the precision and specificity of glutamate-based therapeutics and put research findings into a clinical setting perspective. According to their literature search, glutamate-based therapies act on several brain networks, leading to a rapid antidepressant response by generating rapid alterations in synaptic connectivity. Moreover, they identified relevant biomarkers of rapid antidepressant response, such as altered functional connectivity of limbic, cognitive, and executive nodes.

In their review, Khoodoruth et al. after having examined the role of glutamatergic neurotransmission in the etiopathogenesis of depressive disorders, focused on non-invasive brain stimulation techniques as a potential alternative non-pharmacologic approach to treat depressive symptoms. They stated that the putative cellular mechanisms of action of this group of therapies involve the activity over the glutamatergic neuronal firing, depending on the technique and polarity used. Specifically transcranial magnetic stimulation (TMS) and electroconvulsive therapy (ECT) can depolarize the cell membrane and generate an action potential while transcutaneous direct current stimulation (tDCS) may have an impact on the action potential depending on the polarity. This way, the after-effects are mediated by long-term potentiation (LTP)- like mechanisms where the up-regulation of neurotransmitter release facilitates the opening of AMPARs.

The original research of Yap et al. compared medial prefrontal cortex (MPFC) glutamate levels in healthy perimenopausal (PM) and reproductive-aged (RD) women. The authors performed a Magnetic Resonance Spectroscopy (MRS) study on 15 healthy PM and 16 healthy RD women. The major finding of this study is that MPFC Glu levels were lower in healthy PM women compared to healthy RD women. These findings can be read in light of the context of the debate on perimenopause as a risk factor in the development of depressive symptoms. Women experiencing a premenstrual dysphoric disorder (PMDD) and/or post-partum depression (PPD) are at particular risk of developing MDD during PM. If the correlation between hormonal fluctuations and glutamate alterations in the brain is confirmed in subsequent studies, this could pave the way for the definition of a subtype of depressive disorder more closely linked to hormone-glutamate interactions.

Finally, this collection also covers more clinical aspects. Brandley et al. performed a clinical trial to examine the effects of low glutamate diet on anxiety, post-traumatic stress disorder (PTSD), and depression in 40 veterans with Gulf War Illness (GWI). The diet included nutrients known to be protective against glutamate excitotoxicity, likes omega-3 fatty acids, vitamins B/C/D/E, zinc and magnesium. After 1 month on the low glutamate diet, participants experienced a significant reduction of psychiatric symptoms. Based on these findings, the authors discussed the role of low glutamate diet in combination with an antioxidant-rich diet in the management of psychiatric symptoms.

Overall, this issue covers a broad spectrum of questions relevant for clinical research about the glutamate hypothesis for mood disorders. We hope that this topic collection contributes to the great progress in this field.

## Author contributions

All authors listed have made a substantial, direct, and intellectual contribution to the work and approved it for publication.

## Conflict of interest

The authors declare that the research was conducted in the absence of any commercial or financial relationships that could be construed as a potential conflict of interest.

## Publisher's note

All claims expressed in this article are solely those of the authors and do not necessarily represent those of their affiliated organizations, or those of the publisher, the editors and the reviewers. Any product that may be evaluated in this article, or claim that may be made by its manufacturer, is not guaranteed or endorsed by the publisher.

## References

[1] Salomon RM Miller HL Krystal JH Heninger GR Charney DS. Lack of behavioral effects of monoamine depletion in healthy subjects. Biol Psychiatry. (1997) 41:58–64. 10.1016/0006-3223(95)00670-2 8988796

[2] Hasler G Drevets WC Manji HK Charney DS. Discovering endophenotypes for major depression. Neuropsychopharmacology. (2004) 29:1765–81. 10.1038/sj.npp.1300506 15213704

[3] Moncrieff J Cooper RE Stockmann T Amendola S Hengartner MP Horowitz MA. The serotonin theory of depression: a systematic umbrella review of the evidence. Mol Psychiatry. (2022). 10.1038/s41380-022-01661-0. [Epub ahead of print]. 35854107 PMC10618090

[4] Gaynes BN Lux L Gartlehner G Asher G Forman-Hoffman V Green J. Defining treatment-resistant depression. Depress Anxiety. (2020) 37:134–45. 10.1002/da.22968 31638723

[5] Fava M Davidson KG. Definition and epidemiology of treatment-resistant depression. Psychiatr Clin North Am. (1996) 19:179–200. 10.1016/S0193-953X(05)70283-5 8827185

[6] Abdallah CG Sanacora G Duman RS Krystal JH. Ketamine and rapid-acting antidepressants: a window into a new neurobiology for mood disorder therapeutics. Annu Rev Med. (2015) 66:509–23. 10.1146/annurev-med-053013-062946 25341010 PMC4428310

[7] Kadriu B Farmer CA Yuan P Park LT Deng ZD Moaddel R. The kynurenine pathway and bipolar disorder: intersection of the monoaminergic and glutamatergic systems and immune response. Mol Psychiatry. (2021) 26:4085–95. 10.1038/s41380-019-0589-8 31732715 PMC7225078

[8] Guglielmo R Hasler G. The neuroprotective and neuroplastic potential of glutamatergic therapeutic drugs in bipolar disorder. Neurosci Biobehav Rev. (2022) 142:104906. 10.1016/j.neubiorev.2022.104906 36206993

